# Is tailored treatment superior to non-tailored treatment for pain and disability in women with non-specific neck pain? A randomized controlled trial

**DOI:** 10.1186/s12891-016-1263-9

**Published:** 2016-09-30

**Authors:** Åsa Svedmark, Mats Djupsjöbacka, Charlotte Häger, Gwendolen Jull, Martin Björklund

**Affiliations:** 1 Department of Community Medicine and Rehabilitation, Physiotherapy, Umeå University, SE-901 87 Umeå, Sweden; 2 Centre for Musculoskeletal Research, Department of Occupational and Public Health Sciences, University of Gävle, Gävle, Sweden; 3 Centre of Clinical Research Excellence in Spinal Pain, Injury and Health, The University of Queensland, Brisbane, QLD 4072 Australia

**Keywords:** Neck-shoulder pain, Trapezius, Individualized treatment, Rehabilitation, Physiotherapy, Cut-off

## Abstract

**Background:**

The evidence for the effect of treatments of neck pain is modest. In the absence of causal treatments, a possibility is to tailor the treatment to the individuals’ functional limitations and symptoms. The aim was to evaluate treatment effects of a tailored treatment versus a non-tailored treatment. Our hypothesis was that tailored treatment (TT) would have better effect on pain intensity and disability than either non-tailored treatment (NTT) (same treatment components but applied quasi-randomly) or treatment-as-usual (TAU) (no treatment from the study, no restrictions). We further hypothesized that TT and NTT would both have better effect than TAU.

**Method:**

One hundred twenty working women with subacute and chronic non-specific neck pain were allocated to 11 weeks of either TT, NTT or TAU in a randomized controlled trial with follow-ups at 3, 9 and 15 months. The TT was designed from a decision model based on assessment of function and symptoms with defined cut-off levels for the following categories: reduced cervical mobility, impaired neck-shoulder strength and motor control, impaired eye-head-neck control, trapezius myalgia and cervicogenic headache. Primary outcomes were pain and disability. Secondary outcomes were symptoms, general improvement, work productivity, and pressure pain threshold of m. trapezius.

**Results:**

Linear mixed models analysis showed no differences between TT and NTT besides work productivity favoring TT at 9- and 15-months follow-ups. TT and NTT improved significantly more than TAU on pain, disability and symptoms at 3-month follow-up. General improvement also favored TT and NTT over TAU at all follow-ups.

**Conclusion:**

Tailored treatment according to our proposed decision model was not more effective than non-tailored treatment in women with subacute and chronic neck pain. Both tailored and non-tailored treatments had better short-term effects than treatment-as-usual, supporting active and specific exercise therapy, although therapist-patient interaction was not controlled for. Better understanding of the importance of functional impairments for pain and disability, in combination with a more precise tailoring of specific treatment components, is needed to progress.

**Trial registration:**

Current Controlled Trials ISRCTN 49348025. Registered 2 August 2011.

**Electronic supplementary material:**

The online version of this article (doi:10.1186/s12891-016-1263-9) contains supplementary material, which is available to authorized users.

## Background

The 1-year prevalence of neck pain among workers in the industrialized world varies between 27 and 48 % [1] and the prevalence is higher in women [2, 3]. Women seek care for neck-shoulder pain more frequently with a 5 year cumulative incidence of 29 % compared to 18 % for men in a healthy working population [4]. The risk of developing neck pain is highest in middle age [5, 6].

In most cases the specific cause of neck pain is uncertain [7] and treatment is guided by symptoms and functional impairments. In clinical practice, the normal approach for non-specific neck pain is to assess each patient and tailor treatment and training to individual needs. Yet there is a lack of evidence on how to tailor treatment as efficiently as possible and whether such tailoring is effective [8, 9]. Most trials on non-specific neck pain have focused on interventions of a single type, producing evidence of modest improvement at best [10]. Single treatments trials have included training the deep cervical flexor muscles [11–13], neck muscle strength training [10, 14, 15], manual therapy [10] and eye-head coordination exercises [12, 16]. The heterogeneity of symptoms and functional impairments in non-specific neck pain could be a reason for the weak evidence for single type treatments. Not surprisingly, the best evidence for treatment of chronic non-specific neck pain seems to be with multimodal approaches [10, 14, 17]. Nevertheless, a challenge resides in determining the best combination of treatments for each individual. Better quality clinical decision-making at the individual level could result in improved outcomes of rehabilitation as only treatment components pertinent to the individual would be prescribed [18].

Individualized approaches have been trialed for neck pain [19, 20], but we found no study on the efficacy of tailored treatment that is described well enough to be replicated, i.e., including a detailed clinical treatment decision model. We contend that to improve neck pain treatment outcomes individualized treatments could be prescribed using a detailed decision model. In such a model, the indications for interventions are based on appropriate cut-off values of standardized, reliable and valid assessments. One way to investigate the effect of such an approach is to conduct a randomized trial in which similar treatment components are available but where individuals in one group receive treatment tailored to their needs and the individuals in the other group have non-tailored treatment.

The aim of this study was therefore to evaluate the treatment effects on pain and disability of a tailored treatment versus a non-tailored treatment for women with subacute and chronic non-specific neck pain. Our hypothesis was that tailored treatment based on a clear-cut decision model has better short, intermediate and long-term effects on neck disability and pain than non-tailored treatment (same treatment components but applied to the individuals in a quasi-random way). We also hypothesized that tailored and non-tailored treatment both would have better effects than so called treatment-as-usual (TAU), considering that all included treatment components in the present study have evidence based effects, even if the evidence on treatments for non-specific neck pain is modest.

## Methods

### Trial design

We conducted a single-center, single-assessor blinded randomized controlled clinical trial (RCT). Participants were randomly assigned to one of three groups in a 1:1:1 ratio; tailored (TT), non-tailored (NTT) treatment or TAU. Participants in the TT and NTT groups received treatments two to three times per week for a period of 11 weeks. Evaluation was performed at 3, 9 and 15 months, respectively, after the start of treatment (cf. published study protocol [21] and trial registration ISRCTN 49348025).

### Participants

The study included 120 working women, aged 20–65 years, with non-specific neck pain for a minimum of six weeks. Participants were recruited consecutively via advertisements on web pages and local newspapers.

Eligibility criteria were: pain in the neck-shoulder region, marked as the dominant pain area in a pain drawing [22], Neck Disability Index (NDI) score ≥10 % (more than no disability) and ≤68 % (less than complete disability) [23], self-reported impaired productivity to work (quality and quantity) due to neck symptoms [24] and Swedish speaking. Exclusion criteria were: trauma-related neck pain, cervical radiculopathy or vestibular dysfunction [25, 26], comorbid medical conditions as cancer, type 1 diabetes, heart disease, rheumatic disease including fibromyalgia, anxiety or depression [27], concurrent low back pain [28], temporomandibular disorders [29], surgery or spinal fracture, severely restricted shoulder flexion or cervical range of motion, catastrophizing thoughts or low treatment expectation as assessed from responses to one question from The Pain Catastrophizing Scale [30] and one on treatment expectation of physical therapy, as used by Hill et al. [31] for prediction of outcomes in patients with neck pain.

The study took place in Umeå, Sweden. The intervention period was August 2011 to June 2012. Two clinical settings were used for the interventions and participants chose the most convenient clinic.

### Randomization

For stratification we used minimization [32, 33] to eliminate imbalance regarding age, pain duration, average pain intensity during the last week (Numeric rating scale 0-10, NRS) and disability due to neck pain (NDI). Following baseline measurements and clinical tests, randomization with minimization was performed using a computer program administered by a technician who was not involved in subject recruitment or data collection. To ensure allocation concealment, an independent administrator then informed the participants to which of the three groups they were allocated. The clinicians were informed about the results of the allocation before the first treatment session.

### Decision model for treatment

The purpose of the decision model was to capture specific functional or physical limitations and/or conditions like trapezius myalgia or cervicogenic headache in each individual in order to identify appropriate treatment components. We used cut-off values to indicate dysfunction in each particular test of cervical movement, muscle and sensorimotor function, respectively. These values were based on either empiric published data or on our own reference data from a parallel study on women with non-specific neck pain and a healthy control group [34]. The limit was set at minimum 20 % below reference control values to distinguish between healthy and non-healthy levels since a 20 % difference is considered clinically important [35]. With reference values at hand, the cut-off could be set either to give precedence to a high sensitivity or high specificity. We gave priority to high specificity over sensitivity when deciding cut-off values to avoid false positive outcomes. Further, we considered the relative number of positive tests, predicted by reference data [34], and adjusted cut-off levels to avoid exceeding >40 % of positive tests to keep the decision model diversified.

#### Treatment categories of the model

The decision model included five main treatment categories of specific functional limitations and symptom based conditions: reduction in cervical flexibility, cervical muscle strength-endurance and function, impaired cervical sensorimotor control, trapezius myalgia and cervicogenic headache. The categories 1 to 5 and subcategories are listed in Table 1 and explained in more detail below. The tests used to determine whether or not to assign treatment components to an individual are presented in a supplementary file, see Additional file 1. The categories and treatments were:

**Table 1:** Decision model of treatment for selection of tailored treatment (for details, see Table 2 in Björklund et al [21])

Main category	Test	Cut-off criteria	Treatment component
1. Reduced cervical flexibility	1.1 Upper cervical		
	a) Flexion-extension	a) < 68°	Upper cervical mobilization and ROM treatment and training
	b) Passive rotation in max flexion	b) <32°	
		Qualifier: a) or b)	
	1.2 Lower cervical		
	a) Flexion-extension	a) <17°	Lower cervical mobilization and ROM treatment and training
	1.3 Upper and lower cervical		
	a) Axial rotation	a) <109°	Upper and lower mobilization and ROM treatment and training
2. Impaired cervical muscle strength-endurance and functional strength	2.1 Cranio-cervical flexion (CCF)		
	a) Maximal voluntary contraction (MVC)	a) < 2.5 Nm	CCF-exercises
	b) Endurance (50 % MVC)	b) < 20 s	
		Qualifier: a) or b)	
	2.2 Cervico-thoracic		
	a) Flexion MVC	a) < 40 N	Strength training cervico-thoracic muscles
	b) Extension MVC	b) < 140 N	
		Qualifier: a) or b)	
	2.3 Lifting ability		
	a) C-PILE	a) < 0.12 kg/kg^a^	Strength training of shoulder-arm muscles
	b) Subjective ability to carry and lift	b) ≥ 4 on the scale 1-6…how do you manage to carry/lift?	
		1 = very good; 6 = very bad [51]	
		Qualifier: a) and b)	
3. Impaired sensor-motor control	3.1 Symptoms and activity limitations	Combinations of dizziness, balance disturbances, headache and difficulties to rotate the head [51].	Cervical repositioning/movement control, oculomotor exercises
	3.2 Peak speed of cervical axial rotation	<170°/s	Peak speed training in cervical axial rotation
4. Trapezius myalgia	4.0		
	a) Physiotherapy assessment	a) Criteria according to Ohlsson and coworkers [26] with amendments [25]	EMG-biofeedback training, a progressive program with 8 standardized exercises
	b) PPT upper trapezius muscles	b) < 175 kPa right; < 168 kPa left	
		Qualifier: a) and b)	
5. Cervicogenic headache	5.0		
	a) Physiotherapy assessment	a) Criteria of the Cervicogenic Headache International Study Group [72] with amendment of reduced ROM for upper cervical and palpable upper cervical joint dysfunction [73]	Mobilization of upper cervical, CCF-exercise, endurance training for scapular muscles, postural correction
	b) Range of motion		

*ROM* range of motion, *Nm* newton meter, *Sec* seconds, *N* newton, *C-Pile* cervical progressive isoinertal lifting evaluation test, *PPT* pressure pain threshold, *kPa* kilopascal, *EMG* electromyography

^a^maximal lifted weight/adjusted body weight [74]

1. Reduced cervical flexibility (Table 1: 1.1; 1.2; 1.3): manual therapy including mobilization and range of motion (ROM)-exercises for the upper and/or lower cervical spine. Precise treatment decisions for the individual participant were made by the treating physiotherapist according to manual therapy principles [36].
2. Impaired cervical muscle strength-endurance and functional strength (Table 1: 2.1; 2.2; 2.3): treatment for cranio-cervical muscle impairments (2.1) was a specific exercise program [37, 38] which included endurance, motor control and posture correction training. Treatment for cervical muscle strength and functional capacity (2.2) was high intensive strength training for neck muscles and for lifting capacity (2.3), strength training for shoulder-arm muscles. The strength training programs (2.2; 2.3) were based on established research protocols according to Ylinen et al. [39] and the American College of Sports Medicine [39, 40].
3. Impaired cervical sensorimotor control (Table 1: 3.1; 3.2): treatment for sub-factor 3.1 consisted of two main types of exercises: cervical repositioning/movement control and oculomotor exercises. The program was based on the work of Kristjansson and Treleaven [41, 42] and included a protocol with exercises and progressions for exercise duration, number of repetitions, movement speed and introduction of unstable support. Exercises were set at a challenging level and temporary reproduction of dizziness or visual disturbances was allowed but not reproduction of head or neck pain. Treatment in sub-factor 3.2 focused on improving the ability to perform fast cervical rotations. Some exercises were the same as in 3.1 but exclusive to 3.2 were quick head movements in different planes and trajectory lengths guided by light flashes.
4. Trapezius myalgia (Table 1: 4.0): EMG-biofeedback treatment program for upper trapezius and eight standardized exercises with gradual progression of difficulty level. This was followed by exercises in specific tasks individualized for each subject in the tailored group. The aim of the EMG-biofeedback training program was to increase awareness of muscle tension in the upper trapezius muscles both in resting positions and in static and dynamic tasks. The use of EMG-biofeedback from the trapezius muscle has been shown to reduce excessive muscular activity and pain intensity [43, 44].
5. Cervicogenic headache (Table 1: 5.0): manual therapy including mobilization and ROM exercises for the upper cervical spine, cranio-cervical flexor (CCF) exercises and low-load endurance training for lower trapezius and serratus anterior, as well as correction of scapular posture. The treatment was guided by current best evidence [45, 46].

The treatments in all categories implemented principles of motor learning theories according to Shumway-Cook and Woollacott [47] and had standardized progressions.

#### Rules for the decision model

##### Tailored treatment

The individual treatment for those in the tailored treatment group was constructed a priori from the results of baseline tests. Treatment components were automatically assigned to all impairments that fulfilled cut-off criteria (Table 1). Participants were to receive at least two treatment components. The project group (authors MD, CH, MB) could add a treatment component(s) in either of the three following circumstances: (i) if baseline tests only yielded one or no cut-off values, (ii) if a treatment component was considered contraindicated/unsuitable for an individual (e.g. unforeseeable reasons), (iii) if the two treatment components assigned were judged to be insufficient for the 11-week rehabilitation period, for example, if the two treatment components 1.1 (ROM upper cervical) was assigned together with 1.2 (ROM lower cervical) a further component would be added (refer combinations, Table 2).

**Table 2:** Combinations of components that lead to the addition of a further component

Component 1	Component 2
1.1. Upper cervical ROM	3.2. Peak speed of cervical axial rotation
1.2. Lower cervical ROM	3.2. Peak speed of cervical axial rotation
1.3. Upper and lower cervical ROM, axial rotation	3.2. Peak speed of cervical axial rotation
3.1. Symptoms and activity limitations	3.2. Peak speed of cervical axial rotation
1.1. Upper cervical ROM	1.2 Lower cervical ROM
1.1. Upper cervical ROM	1.3. Upper and lower cervical ROM, axial rotation
1.2. Lower cervical ROM	1.3. Upper and lower cervical ROM, axial rotation
1.1. Upper cervical ROM	1.3. Upper and lower cervical ROM, axial rotation
+1.2. Lower cervical ROM	

*ROM* range of motion

The decision for which component(s) to add was based on the relative closeness to cut-off for each test as well as the outcome of a structured interview using the Problem Elicitation Technique (PET) [48]. A treatment component could be added if the PET interview clearly indicated specific problems for the participant, for example complaints of difficulties to lift and carry, in combination with a close to cut-off value for the baseline test of lifting capacity. If the participant was presented with cervicogenic headache or trapezius myalgia, she was assigned specific treatment pre-decided components (see treatment categories of the model). Finally, participants could be assessed by an optician if they regularly performed visually demanding near-work. Detailed rules for this assessment are provided in the protocol article [21].

##### Non-tailored treatment

Each participant received two treatment components, in a quasi-random manner, from those not indicated from cut-off values for impairments at baseline assessment. Thus, treatment components specifically targeted to impaired function were not assigned to NTT participants. After excluding treatments indicated by the decision model for that participant, the participant was assigned the next two treatment components (in order) from the residual list.

### Intervention procedures

Written instructions were provided for each treatment component about performance and progressions. Four physiotherapists provided the treatments. All were experienced in treating musculoskeletal disorders and had special competence in manual therapy (two with more than 15 years of work experience, two with 3–7 years of experience). They received 12 h of preparation sessions prior to the study for familiarization with the trial procedures. The physiotherapists treated participants in both TT- and NTT-groups.

#### Tailored treatment

Each participant received the assigned treatments by one physiotherapist. In the latter half of the intervention period, the treatment program was complemented with functional training of daily activities, relevant to the participant’s individual needs as determined in the PET interview. The functional training followed principles of motor learning using for example, external feedback, task variation, training in different contexts with increasingly more complex movement tasks. The purpose of functional training was to promote retention and transfer of new skills obtained through the assigned treatments.

#### Non-tailored treatment

Each participant was given the non-indicated but still established neck pain treatment by one physiotherapist. In the latter half of the intervention period the treatment component program was complemented with functional training in which participants followed a set training program with complex movement exercises called “Muscle Action Quality” (MAQ) training [49]. It included general fitness qualities as strength, flexibility, and balance and movement control. The purpose with the set training program was to add functional training, as in TT group, but without individual adjustment.

#### Treatment-as-usual

Participants randomized to TAU did not receive any treatments within the study and there were no restrictions regarding what treatment they sought (if any). If participants sought health care during the intervention period, it was reported in follow-up questionnaires in a similar manner to the TT and NTT groups.

### Outcomes

The primary outcome measures were: neck disability (Neck Disability Index, NDI% [23]), and average pain intensity last week (0–10 Numeric Rating Scale, NRS [50]). The secondary outcomes included: (i) General improvement, measured with the Patient Global Impression of Change scale (PGICS) [50] which is a single question asking the participants for an estimation of change compared to before the intervention with a 7-point response Likert scale from 1. “very much improved” to 7. “very much worse” (ii) Intensity and frequency of symptoms, measured with the symptom scale, intensity and frequency indices, of the neck specific Profile Fitness Mapping neck questionnaire (ProFitMap-neck) [51]. The index scores are normalized 0-100 with higher scores reflecting less symptoms/better health. (iii) Self-reported work productivity loss, measured with questions of the impact of neck symptoms on the quality and quantity of performed work the latest six weeks [24] (response scale 0-10, 10 equals working as usual) and finally (iv) Pressure pain threshold (PPT) of m. trapezius, assessed with pressure algometer (unit, kPa). Any adverse events were recorded by the intervention leaders and reported to the project leader (MB) for documentation. If a participant in either the TT- or NTT- group experienced an acute pain episode during the intervention that did not settle within a week, the physiotherapist was permitted to assess and treat the problem with manual therapy, for a maximum of three sessions, to reduce the pain. It was considered unethical to withhold a treatment with proven effectiveness for neck pain [14]. This occurred only once during the intervention. The participant in question received three sessions of manual therapy and subsequently, the prescribed intervention could be carried through.

### Sample size

Power calculations for treatment effects of neck disability (NDI), and average pain intensity in the last week (NRS) were performed with a one-way analysis of variance (ANOVA) (nQuery Advisor 3.0). Reference data from a parallel clinical trial [52] showed that the NDI standard deviation (SD) was 10.3 % (based on 117 women with neck-shoulder pain). The clinically important difference for the NDI is considered between 6 and 10 % [53] Given a difference on NDI of 6 % between any of the three groups, power of 0.8 required a minimum of 20 participants per group (alfa = 0.05). The smallest clinical important pain reduction in NRS is approximately 15 % [35]. In the parallel clinical trial, the SD was 15.5 %. Given these facts, 20 participants per group yield a power of >0.8 for a NRS difference of 15 % between any of the three groups (alfa = 0.05). We were conservative and recruited 40 participants per group, to account for any loss to follow-up and to improve the robustness of results.

### Blinding

The researcher conducting all outcome assessments was blinded to group allocation of participants, but it was not possible to blind the treating physiotherapists. Care was taken to conceal the study hypotheses from the participants as well as any clues to their allocation to tailored or non-tailored treatment.

### Statistical methods

The pre-defined hypotheses with its pre-specified between-group contrasts were tested with linear mixed-effects models, a method of analysis that has advantages to handle individual variances and missing values [54, 55]. Analyses were based on intention-to-treat principles meaning that each participant’s available data were used accordant with original allocation and irrespective of the level of attendance. Q-Q plots of residuals were observed to verify that they were not deviated greatly from normal distribution. To evaluate treatment effects, separate models for each primary and secondary outcome were made with independent fixed factors *time* (3, 9 and 15 month after start of intervention, baseline was reference) and *group* (TT, NTT, TAU). Participants were included in the analysis model as a random effect. Least square group means were estimated from the models and change in outcome variables from baseline to follow-up was calculated. Treatment effect was defined as the differences between group changes. The randomization with minimization assured balance between groups on the potential confounder age, pain duration, pain intensity and disability. No further adjustment was done in the analysis. Group means, standard deviation (SD), effects and 95 % confidence intervals (CI) are presented. All analyses were conducted using the statistical computing program R [56] and linear mixed-effects models were fitted using the R package LME4 [57]. The level of significance was set at alpha level 0.05.

## Results

### Recruitment and participants

**Fig. 1:**
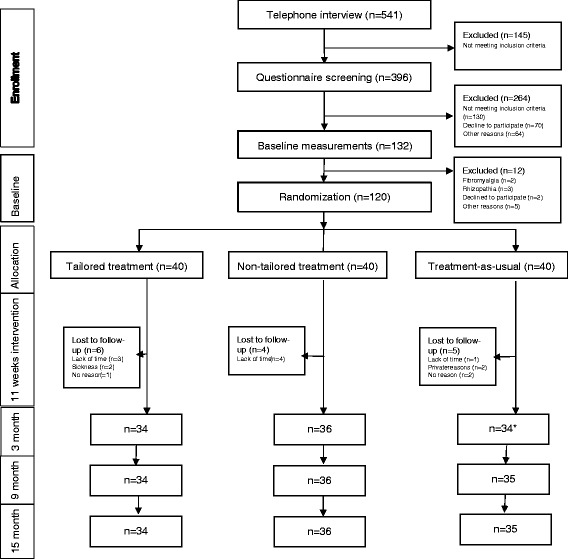
Flow diagram of the recruitment, group allocation and participation in the study.*One participant forgot to answer questionnaire at 3 month follow-up. All participants (*n* = 120) were included in the analysis

**Fig. 2:**
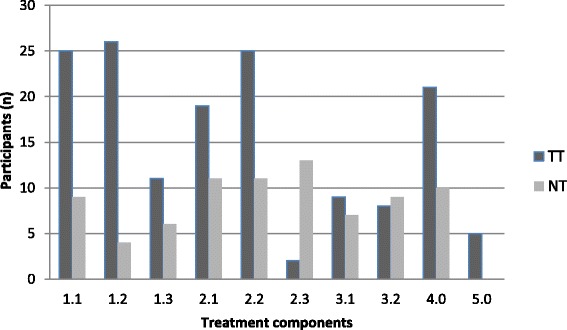
Distribution of treatment components for participants in tailored treatment (TT) group (*n* = 40) and non-tailored treatment (NTT) group (*n* = 40) group; 1.1: Upper cervical mobilization, 1.2: Lower cervical mobilization, 1.3: Upper and lower cervical mobilization, 2.1: Cranio-cervical flexion-exercises, 2.2: Strength training cervico-thoracic muscles, 2.3: Strength training shoulder-arm muscles, 3.1: Cervical repositioning/movement control, oculomotor exercises, 3.2: Peak speed training in cervical axial rotation, 4.0: Trapezius myalgia, EMG biofeedback training, 5.0: Cervicogenic headache training program

Recruitment started in June 2011 and ended in March 2012 when all required participants had entered the study. A total of 541 participants were assessed for eligibility and 120 of those were consecutively randomized to one of the three groups (Fig. 1). During the intervention period, six participants from the TT- group, four from the NTT-group and six from the TAU-group dropped out and one participant failed to complete the questionnaires after intervention but undertook all other assessments at all test events. Thirty-four participants completed all treatment sessions in the TT-group while 36 did so in the NTT-group. Table 3 presents the baseline demographics and clinical characteristics of participants. Use of other health care, work absence days and physical activity level for all groups are described in Table 4. Distribution of treatment components is illustrated in Fig. 2. Total number of treatment components given was ruled by the decision model with two or more treatments per person in the TT-group and two treatments per person in the NTT-group. Compared to the TT-group, treatment components were more evenly distributed in the NTT-group and strength training for shoulder-arm muscles (2.3) was considerably more prevalent in the NTT-group. An adverse event was reported by one NTT-group participant who experienced arm pain after the intervention.

**Table 3:** Baseline demographics and clinical characteristics of participants in the randomized groups

Demographics	Tailored treatment (*n* = 40)	Non-tailored treatment (*n* = 40)	Treatment-as-usual (*n* = 40)
Age, years, mean (SD)	47 (11.2)	48 (12.6)	47 (11.1)
Weight, kg, mean (SD)	68.9 (12.5)	66.8 (10.5)	68 (14.4)
BMI, kg/m^2^, mean (SD)	24.7 (4)	24.5 (3.7)	24.8 (4.9)
Use of tobacco (n); smoker/snuffer^a^	1/3	1/4	1/8
Physical activity, leisure^b^ median (IQR)	5 (4-5)	4 (4-4.5)	5 (4.5-5)
Physical activity, work^c^ median (IQR)	2 (1.5-2)	2 (1.5-2.5)	1 (1-1.5)
Duration of pain, month, median (IQR)	63.5 (24-144)	66 (24-150)	60 (23.5–120)

*BMI* body mass index; ^a^ snuff is a small portion of tobacco used under the upper lip: ^b^ Physical activity, leisure, scale 1-6: 1-2 = low, 3-4 = medium, 5-6 = high [75]; ^c^ Physical activity, work, scale 1-4: 1 = mostly sitting work, 2 = light physical work, 3 = quite physically exhausting work, 4 = very physically exhausting work

**Table 4:** Health care use and work absence days between baseline and three month follow-up for the 3 randomized groups

	Tailored treatment (*n* = 34)	Non-tailored treatment (*n* = 36)	Treatment-as-usual (*n* = 33)
Participants searched care	4	6	16
Work absence days (n)	2 (1)	5 (3)	7 (4)
# Visits (participants):			
Medical doctor	1 (1)	1 (1)	1 (1)
Physiotherapist		8 (2)	46 (9)
Naprapath		5 (2)	2 (1)
Masseur	7 (3)	16 (4)	21 (7)
Total # Visits (participants)	8 (4)	30 (6)	70 (16)

### Primary outcomes

We found no significant difference in treatment effects between TT and NTT on primary outcomes in any of the three follow-up evaluations (Table 5).

**Table 5:** Descriptive statistics of primary outcome measured at baseline, 3-month, 9-month and 15-month after start of intervention and treatment effects as changes from baseline compared between groups

	TT	NTT	TAU	TT vs NTT	TT vs TAU	NTT vs TAU
	Estimated mean (SD)	Estimated mean (SD)	Estimated mean (SD)	Effects^b^ (95 % CI)	Effects^b^ (95 % CI)	Effects^c^ (95 % CI)
NRS						
Baseline	4.55 (2.08)	4.57 (2.08)	4.72 (2.08)			
3 month	2.62 (2.04)	2.56 (2.04)	3.97 (2.04)	−0.09 (-0.48 to 1.5)	1.17 (0.15 to 2.19)*	1.26 (0.27 to 2.25)*
9 month	2.85 (2.04)	3.31 (2.04)	3.5 (2.07)	0.43 (-1.55 to 2.41)	0.47 (-0.52 to 1.46)	0.04 (-0.95 to 1.03)
15 month	3.06 (2.04)	2.85 (2.04)	3.6 (2.07)	−0.24 (-2.22 to 1.74)	0.41 (-0.58 to 1.4)	0.65 (-0.34 to 1.64)
NDI						
Baseline	21.84 (10.18)	24.39 (10.18)	24.18 (10.05)			
3 month	14.62 (9.85)	14.39 (9.96)	21.74 (9.93)	−2.78 (-6.85 to 1.29)	4.78 (0.67 to 8.89)*	7.56 (3.49 to 11.63)***
9 month	14.84 (9.85)	16.61 (9.96)	19.65 (9.93)	−0.78 (-4.87 to 3.31)	2.47 (-1.64 to 6.58)	3.25 (-0.82 to 7.32)
15 month	15.20 (9.85)	13.80 (9.96)	17.99 (9.94)	−3.95 (-8.02 to 0.12)	0.45 (-3.64 to 4.54)	4.40 (0.35 to 8.45)*

*TT* tailored treatment, *NTT* non-tailored treatment, *TAU* treatment as usual group, *vs* versus, *SD* standard deviation, *CI* confidence interval, *NRS* Average pain intensity last week (0-10), *NDI* neck disability index (0-100); ^b^ Positive values of effects favor the tailored group; ^c^ Positive values of effects favor the non-tailored group; * comparison is significant at the 0.05 level. ** comparison is significant at the 0.01 level. *** comparison is significant at the 0.001 level

Compared with TAU, TT and NTT showed significant treatment effects at 3-month follow-up with improved neck disability (absolute within-group differences NDI%: TT, -7 ; NTT, -10 ; TAU, -2,5) and reduced average pain intensity last week (absolute within-group differences NRS points: TT, -1,9 ; NTT, -2,0 ; TAU, -0,75) (Table 5). At the 9-month follow-up, there were no differences between groups. At the 15-month follow-up, neck disability of the NTT-group was significantly improved compared with the TAU-group but not compared with the TT-group (absolute within-group differences NDI%: TT,-6,5 ; NTT, -10,5 ; TAU, -6 ).

### Secondary outcomes

Descriptive group statistics and treatment effect of secondary outcome measures are shown in Table 6. At the 9-month follow-up, the TT-group showed significantly improved quality of performed work compared with the NTT-group. After 15 month, improvement in both quality and quantity of performed work was significantly greater in the TT-group compared with the NTT-group.

**Table 6:** Descriptive statistics of secondary outcome measured at baseline, 3 month, 9-month and 15-month after start of intervention and treatment effects as changes from baseline compared between groups

Secondary Outcomes	TT	NTT	TAU	TT vs NTT	TT vs TAU	NTT vs TAU
	Estimated mean (SD)	Estimated mean (SD)	Estimated mean (SD)	Effects^b^ (95 % CI)	Effects^b^ (95 % CI)	Effects^c^ (95 % CI)
ProFitMap intensity						
Baseline	71.64 (11.38)	71.55 (11.38)	70 (11.38)			
3 month	79.61 (11.02)	80.84 (11.16)	72.07 (11.07)	−1.31 (-3 to 5.62)	5.9 (1.51 to 10.29)**	7.21 (2.88 to 11.54)**
9 month	78.48 (11.07)	81.49 (11.16)	75.91 (11.24)	−3.09 (-1.22 to 7.40)	0.92 (-3.49 to 5.33)	4.01 (-0.32 to 8.34)
15 month	78.5 (11.02)	82.1 (11.22)	76.98 (11.18)	−3.67 (-0.64 to 7.98)	−0.13 (-4.48 to 4.22)	3.54 (-0.79 to 7.87)
ProFitMap frequency						
Baseline	66.02 (14.67)	67.74 (14.67)	64.08 (14.67)			
3 month	78.19 (14.05)	78.69 (14.22)	67.28 (14.16)	1.22 (-3.91 to 6.35)	8.97 (3.74 to 14.2)***	7.73 (2.56 to 12.9)**
9 month	75.74 (14.05)	76.09 (14.28)	71.44 (14.25)	1.38 (-3.75 to 6.51)	2.36 (-2.85 to 7.57)	0.98 (-4.19 to 6.15)
15 month	75.76 (14.05)	78.73 (14.34)	72.88 (14.25)	−1.24 (-6.41 to 3.9 )	0.95 (-4.26 to 3.61)	2.19 (-2.98 to 7.36)
PGICS						
3 month	2.20 (1.1)	2.19 (1.14)	3.64 (1.1)	0.01 (-0.52 to 0.54)	1.44 (0.9 to 1.98)***	1.45 (0.93 to 1.97)***
9 month	2.5 (1.16)	2.53 (1.14)	3.13 (1.18)	0.04 (-0.49 to 0.57)	0.8 (0.31 to 1.29)**	0.85 (0.38 to 1.32)***
15 month	2.49 (1.16)	2.47 (1.14)	3.18 (1.18)	−0.005 (-0.48 to 0.46)	0.75 (0.28 to 1.22)**	0.75 (0.28 to 1.22)**
PPT right						
Baseline	223.4 (102.7)	209.8 (102.8)	209.2 (102.8)			
3 month	251.6 (99.5)	237 (100.4)	203.3 (100.2)	1.03 (-38.4 to 40.4)	34.1 (-6.1 to 74.3)	33.06 (-6.6 to 72.8)
9 month	251.5 (100.1)	263 (100.5)	228.4 (102.3)	−25.10 (-65.7 to 14.5)	8.92 (-31.4 to 49.3)	34.03 (-5.7 to 73.7)
PPT left						
Baseline	218.1 (95.8)	204.9 (95.8)	212.3 (95.8)			
3 month	247.6 (93.3)	223.4 (94.1)	197.3 (94.2)	11.01 (-28.8 to 50.9)	44.49 (3.7 to 85.2)*	33.47 (-6.6 to 73.6)
9 month	244.8 (94.1)	243.7 (93.5)	224.6 (95.6)	−11.99 (-52.1 to 28.1)	14.46 (-26.3 to 55.2)	26.45 (-13.65 to 66.6)
Work Quantity						
Baseline^d^	8.51 (1.58)	8.79 (1.58)	7.95 (1.51)			
3 month	9.23 (1.63)	9.15 (1.56)	8.91 (1.51)	0.36 (-0.46 to 1.18)	−0.25 (-1.07 to 0.57)	−0.61 (-1.47 to 0.19)
9 month	9.58 (1.63)	9.24 (1.56)	8.94 (1.53)	0.62 (-0.2 to 1.44)	0.07 (-0.75 to 0.89)	−0.55 (-1.16 to 0.25)
15 month	9.75 (1.63)	9.09 (1.56)	9.5 (1.53)	0.94 (0.12 to 1.76)*	−0.32 (-1.14 to 0.5)	−1.26 (-2.06 to -0.46)**
Work Quality						
Baseline^e^	7.43 (1.83)	8.32 (1.89)	7.53 (1.86)			
3 month	8.77 (1.86)	9.06 (1.8)	8.58 (1.74)	0.61 (-0.31 to 1.53)	0.28 (-0.64 to 1.2)	−0.32 (-1.22 to 0.58)
9 month	9.18 (1.86)	9.0 (1.8)	8.52 (1.77)	1.08 (0.16 to 2)*	0.76 (-0.16 to 1.68)	−0.32 (-1.22 to 0.58)
15 month	9.36 (1.86)	9.09 (1.8)	9.11 (1.77)	1.17 (0.25 to 2.09)*	0.34 (-0.58 to 1.26)	−0.82 (-1.72 to 0.08)

*TT* tailored treatment group, *NTT* non-tailored treatment group, *TAU* treatment as usual group, *vs* versus, *SD* standard deviation, *CI* confidence interval; *PGICS* patient global impression of change scale, *PPT* pressure pain threshold; ^b^ Positive values of effects favor the tailored treatment group; ^c^ Positive values of effects favor the non-tailored treatment group; ^d^ TAU, significant lower (*p* < 0,05) than NT; ^e^ TT, significant lower (*p* < 0,05) than NTT; * comparison is significant at the 0,05 level, ** comparison is significant at the 0,01 level, *** comparison is significant at the 0,001 level

In comparison with the TAU-group, the TT- and the NTT- groups reduced intensity and frequency of symptoms (ProFitMap-neck) and showed higher general improvement (PGICS) at the 3-month follow-up. Also pressure pain threshold (PPT) on the left m. trapezius was increased in the TT-group compared with the TAU-group at the same follow-up. At 9- and 15- month follow-ups, the TT- and NTT-groups again rated their general improvement higher than the TAU-group. At the 15-month follow-up the TAU-group improved in work productivity (quantity of performed work) compared with the NTT-group.

## Discussion

Our first hypothesis, that women with neck pain would benefit more from TT than NTT based on a decision model with a specific test battery covering common problems for neck patients, was rejected. The only outcome to favor TT over NTT was improvement in work productivity, at both 9- and 15-month follow-up. Our second hypothesis of superior effects of any treatment was supported since both intervention groups improved significantly more than the control group.

This is a first attempt to structure and implement a concise decision model for neck rehabilitation, where treatment choices for individuals with non-specific neck pain are based on cut-off levels in specific tests. We have found only one other study that has implemented a decision model to individualize treatment for neck pain [20]. In contrast to our model with cut-off levels and a structured interview, Wang et al used a clinical reasoning algorithm developed by an experienced physiotherapist. Although their study favored the individual approach, the absence of a treatment control group and long-term follow-up reduces the impact of their findings. Other studies evaluating tailored interventions compared with general approaches for neck pain did not include decision models on which the tailoring of interventions is founded [19, 58, 59].

### Tailored versus Non-tailored treatment

The only difference found between TT and NTT was in the secondary outcome measure, work productivity loss. In contrast to NTT, the TT included functional training of limitations of daily activities, as identified in the PET interview, guided by principles of motor learning. This may have mattered for the work productivity result. The difference between groups was nevertheless small and a regression-towards-mean effect cannot be disregarded, taking into account the significantly lower baseline value of the quality of work for the TT-group. The fact that the TAU-group also showed improved work productivity at 15-months compared with the NTT-group further reduces the significance of this finding.

There could be various explanations for why TT was not superior to NTT. Firstly, there may be inadequacies in our model to guide the prescription of TT. The model may not be sensitive enough to sufficiently separate indicated or non-indicated treatment components, which could lead to equal effects on targeted functions in both groups. We chose cut-off values for impairments based on empiric data from the literature and our own reference data [34]. We tried to determine a high level of specificity, where no more than 40 % of participants would receive the same treatment in order to keep the model diversified. This attempt failed on four treatment components where more than 50 % of participants in TT-group needed these treatments according to the model (Fig. 2). On the other hand, cut-off level for the shoulder-arm strength training (Table 1, 2.3), based on the lifting test C-PILE, was clearly too low for our sample with the consequence that only two participants in the TT-group (compared to 13 in the NTT-group, Fig. 2) qualified as “impaired” and received this component. This cut-off value was based on the only reference data available for the C-PILE test [60]. The sample in that study had clearly higher disability and pain compared with our participants, which possibly confounded our prescription of this treatment. The limited available evidence of certain tests of functioning in neck pain demonstrates the difficulty to individualize treatment based on decision tools with clear cut-off values. Other reasons for the indifferent result between TT and NTT may be connected with the treatment components and the targeted functional limitations of the model. Tailoring with the treatment components used in this study may simply not be of importance for the targeted population. The selected treatment components show, at best, moderate evidence of effect in chronic non-specific neck pain [10]. Also, improved function may not be strongly associated with decreased self-rated pain and disability. Steiger and coworkers [61] reviewed the association between changes in pain and disability with changes in targeted aspects of physical function in treatment of chronic non-specific low back pain and found little evidence to support an association. The same relationship is poorly investigated in people with neck pain and our study design with individualized and diversified treatment prescriptions does, unfortunately, not permit deeper analyses of this topic.

A design issue related to difficulties using cut-off levels concerns the two allocated treatment components that participants in NTT-group received. In instances when the NTT participant’s test result for the allocated treatment was just above cut-off level, there was a chance that a treatment which was actually indicated was given to the participant. Perhaps evaluation of tailored versus non-tailored treatment would be better served with designs that increase the contrast between TT-and NTT-group interventions. For example, to allocate two treatment components with test results farthest from cut-off level in each individual case in the NTT-group to ensure non-indicated management. Such a design would, however, probably not live up to a realistic clinical situation.

### Tailored and non-tailored treatment versus treatment-as-usual

The TT- and the NTT-groups improved more than the TAU-group in the short-term. This effect of TT and NTT, both including active and specific exercise therapy, corroborates earlier findings [10, 62]. Our results are also in line with the systematic review by Gross et al [63] that concluded that there was no difference in pain outcomes between various exercise approaches for persons with neck pain of mechanical origin. The similarity in outcome between TT and NTT may partly be explained by the common effect of movement and exercise in stimulating peripheral and central mechanisms which modulate pain [64–66]. The likely placebo effect from the therapist-participant interaction [67] in the TT and NTT can also play a part of the superiority of these interventions relative to TAU.

In our study there were long-term effects on general improvement for the TT- and NTT-groups, and on neck disability for NTT, compared with TAU. Long-term treatment effects in chronic neck pain are rare. However, positive long-term benefits for physical function may be present when active treatment is compared to less active treatment, and if home exercises are maintained [68]. Participants in our study received home exercises if prescribed by the physiotherapists, but it was not systematically evaluated.

### Study strength and limitations

A strength of our study is the low attrition rate, only 12 % drop outs. The lack of high quality evidence for effective treatments for chronic non-specific neck pain [63] could obviously be seen as a general limitation of the study, but current best evidence treatments were used. Even though our sample was well defined, it can be seen as a convenience sample thus lowering the generalizability of the results to women with non-specific neck pain. It is also likely that this population is heterogeneous with respect to etiology, but our decision model, treatments and outcomes focused mainly on the physical dimension. Regarding confounders, the study design can be considered a strength of the study with the randomization by minimization providing equal distributions of age, pain level, pain duration and neck disability. Some predictors of poor outcomes, catastrophizing, anxiety and depression [31], were exclusion criteria in an attempt to delimit the study to avoid the decision model from becoming too diversified and complex. Nevertheless, psychosocial factors may still have influenced the results. We did not control for stress, perceived muscular tension, psychosocial factors at work, type of work and work hours that could have influenced the neck pain [69–71].

Further limitation concerns our individualized study design that did not permit a clear evaluation of possible intermediate effects on functioning targeted by the decision model.

### Future research

The results of this study may be a consequence of the decision model lacking precise enough cut-off levels, or that associations between changes in the targeted functions of the model and pain/disability are too weak. Further research into the effects of tailoring interventions for neck pain disorders is warranted, but in the first instance more precise knowledge is needed regarding cut-off levels to determine impairment if the current decision-model is to be further developed.

## Conclusions

This RCT found no support for tailored over non-tailored treatment of women with subacute or chronic non-specific neck pain when interventions were prescribed according to a decision model that used cut-off levels in various functional tests. Nevertheless, the evidence based treatment components incorporated in our model, regardless of intervention group, resulted in better short-term effects than treatment-as-usual, albeit that the therapist-participant interaction was not controlled for.

## Additional file

Additional file 1: Supplementary appendix. (PDF 397 kb)

## Abbreviations

ANOVA: Analysis of variance
CCF: Cranio-cervical flexion
C-PILE: Cervical progressive isoinertial lifting evaluation test
EMG: Electromyography
kPa: Kilopascal
MAQ: Muscle action quality
MVC: Maximal voluntary contraction
NDI: Neck disability index
NRS: Numeric rating scale
NTT-group: Non-tailored treatment group
PET: Problem elicitation technique
PGICS: Patient Global Impression of Change Scale
PPT: Pressure pain threshold
ProFitMap-neck: Profile Fitness Mapping neck questionnaire
RCT: Randomized controlled clinical trial
ROM: Range of motion
TAU: Treatment-as-usual
TT-group: Tailored treatment group
