# Supplementary material for: Is tailored treatment superior to non-tailored treatment for pain and disability in women with non-specific neck pain? A randomized controlled trial
Source: BMC Musculoskelet Disord. 2016 Sep 30;17:408. doi: 10.1186/s12891-016-1263-9 (PMC5045621; doi:10.1186/s12891-016-1263-9)
Supplement: Additional file 1: — Supplementary appendix. (PDF 397 kb) [file 12891_2016_1263_MOESM1_ESM.pdf]

APPENDIX:

Baseline assessment and test of function

Cervical flexibility (1.1; 1.2; 1.3) was tested in a seated position with the upper body 4-point belted and eyes closed as described by Rudolfsson et al. [1]. An electromagnetic tracking system (FASTRAK®, Polhemus Inc.) was used to measure cervical sagittal extension and flexion from a neutral head position, axial rotation and a combined movement of axial rotation in a maximal forward neck flexion position (degrees). Range of motion (ROM) in the upper and lower cervical parts during extension and flexion were recorded separately [1].

Cervical muscle strength-endurance and functional strength (2.1; 2.2; 2.3) were tested in three different tasks. First, cranio-cervical flexor (CCF) isometric strength and endurance (2.1) were tested in a standing position with the body secured by wide strips around chest and waist. An application pad was positioned under the mandible and participants were instructed to push against the pad as if performing a "nod" with the head. Torque was recorded in newton-meters (Nm). The protocol was based on that of O´Leary et al [2] in conformity with Van Wyje el al [3]. Second, isometric strength of the cervical flexors and extensors (2.2) was tested according to Salo et al [4] with participants in a seated position with wide strips around chest and waist. A bar with a force cell was placed against the participant's forehead for flexion strength and against the occiput for extension strength, providing values in Newton (N). Third, functional strength (2.3) was measured by a dynamic lifting task from waist to shoulder height, the Cervical Progressive Isoinertial Lifting Evaluation test (C-PILE) [5]. Maximal lifting capacity was measured in maximal lifted kilogram divided with participant weight. In order to minimize the effects of participant´s body under or over weights, a formula called "adjusted weight" according to Mayer at al [5] was used. In the subcategory 2.3, the participants also provided a subjective rating of their ability to carry and to lift.

Cervical sensorimotor control (3.1;3,2), the first subcategory 3,1 was evaluated by questions about dizziness, balance disturbances and difficulties to rotate the neck, due to neck problems [6], and neck related headache. The second subcategory 3,2 was a test of maximal speed of cervical axial rotation measured in degrees per second. This was performed in same setup as test of cervical flexibility and measured by FASTRAK® [7]. The subject was sitting with the head in a neutral position waiting for a sound that indicated a fast axial rotation to the right or left.

Trapezius myalgia (4.0) was diagnosed with the criteria neck pain, tightness of the trapezius muscle and palpable tender points in the trapezius muscle [8, 9]. Pressure pain thresholds (PPT) were measured using an algometer to quantify muscle tenderness (kPa).

Cervicogenic headache (5.0) diagnosis was based on the major criteria of Cervicogenic Headache International Study Group [10] combined with reduced ROM in the upper cervical spine and palpable upper cervical joint dysfunction [11].

1. Rudolfsson T, Bjorklund M, Djupsjobacka M: **Range of motion in the upper and lower cervical spine in people with chronic neck pain**. *Man Ther* 2012, **17**(1):53-59.
2. O'Leary SP, Vicenzino BT, Jull GA: **A new method of isometric dynamometry for the craniocervical flexor muscles**. *Phys Ther* 2005, **85**(6):556-564.
3. Van Wyk L, Jull G, Vicenzino B, Greaves M, O'Leary S: **A comparison of craniocervical and cervicothoracic muscle strength in healthy individuals**. *J Appl Biomech* 2010, **26**(4):400-406.
4. Salo PK, Ylinen JJ, Malkia EA, Kautiainen H, Hakkinen AH: **Isometric strength of the cervical flexor, extensor, and rotator muscles in 220 healthy females aged 20 to 59 years**. *J Orthop Sports Phys Ther* 2006, **36**(7):495-502.
5. Mayer TG, Barnes D, Kishino ND, Nichols G, Gatchel RJ, Mayer H, Mooney V: **Progressive isoinertial lifting evaluation. I. A standardized protocol and normative database**. *Spine (Phila Pa 1976)* 1988, **13**(9):993-997.
6. Bjorklund M, Hamberg J, Heiden M, Barnekow-Bergkvist M: **The ProFitMap-neck--reliability and validity of a questionnaire for measuring symptoms and functional limitations in neck pain**. *Disabil Rehabil* 2012, **34**(13):1096-1107.
7. Röijezon U, Djupsjobacka M, Bjorklund M, Hager-Ross C, Grip H, Liebermann DG: **Kinematics of fast cervical rotations in persons with chronic neck pain: a cross-sectional and reliability study**. *BMC Musculoskelet Disord* 2010, **11**:222.
8. Ohlsson K, Attewell RG, Johnsson B, Ahlm A, Skerfving S: **An assessment of neck and upper extremity disorders by questionnaire and clinical examination**. *Ergonomics* 1994, **37**(5):891-897.
9. Juul-Kristensen B, Kadefors R, Hansen K, Bystrom P, Sandsjo L, Sjogaard G: **Clinical signs and physical function in neck and upper extremities among elderly female computer users: the NEW study**. *Eur J Appl Physiol* 2006, **96**(2):136-145.
10. Sjaastad O, Fredriksen TA, Pfaffenrath V: **Cervicogenic headache: diagnostic criteria. The Cervicogenic Headache International Study Group**. *Headache* 1998, **38**(6):442-445.
11. Jull G, Amiri M, Bullock-Saxton J, Darnell R, Lander C: **Cervical musculoskeletal impairment in frequent intermittent headache. Part 1: Subjects with single headaches**. *Cephalalgia* 2007, **27**(7):793-802.
